# The Hannover experience: Surgical treatment of tongue cancer - A clinical retrospective evaluation over a 30 years period

**DOI:** 10.1186/1758-3284-3-27

**Published:** 2011-05-21

**Authors:** Horst Kokemueller, Majeed Rana, Jennifer Rublack, Andre Eckardt, Frank Tavassol, Paul Schumann, Daniel Lindhorst, Martin Ruecker, Nils-Claudius Gellrich

**Affiliations:** 1 Department for Oral and Maxillofacial Surgery, Hannover Medical School Carl-Neuberg-Str. 1, 30625 Hannover, Germany

**Keywords:** tongue cancer, squamous cell carcinoma, resection, survival, prognostic factors

## Abstract

**Objectives:**

In this retrospective study, we present a clinical review of our experience with tongue cancer in order to obtain valid criteria for therapeutic decision-making.

**Materials and methods:**

Between 1980 and 2009, a total of 341 patients with squamous cell carcinoma of the tongue were treated at our Department. The average follow-up was 5.2 years. 309 patients received surgical treatment, which was combined in nearly 10% with neoadjuvant and in nearly 20% with postoperative radio(chemo)therapy. 32 patients were excluded from surgery and received primary radiation.

**Results:**

Local and regional failure occurred in 23.9% and 20.4%, leading to a total failure rate of 37.2% after an average duration of 1,6 years. N-Status, extracapsular spread and clear margins were identified as the dominant factors for survival, which was calculated with 54.5% after 5 years.

**Conclusions:**

We recommend categorical bilateral neck dissection in order to reliably remove occult lymph node metastases. Adjuvant treatment modalities should be applied more frequently in controlled clinical trials and should generally be implemented in cases with unclear margins and lymphatic spread.

**Clinical relevance:**

This study provides new treatment strategies for primary tumour disease and for tumour recurrence.

## Introduction

Tongue cancer is the most common malignancy diagnosed within the oral cavity, which accounts between 25 and 40% of oral squamous cell carcinomas [1]. Despite the development of multimodal treatment options, the prognosis remains relatively poor. Manifest and occult lymph node metastasis are observed more often than in any other cancer of the oral cavity [2]. The tongue seems to be predisposed for malignant invasion due to its highly muscularized structure and its rich lymphatic network [3]. Extensive resection with implementation of elective neck dissection especially in early stages of tongue cancer has therefore been a source of debate in recent years [4-6].

Only a limited number of studies have examined larger series of tongue cancer. Spiro and Strong evaluated 314 patients (1957-1963) with tongue cancer and found an overall 5-year survival rate of only 42% [7]. In a later study from the same institution with 412 patients (1969-1978), Callery et al. noted an increased proportion of female patients and an increased involvement of the base of tongue compared to the earlier decade [8]. More patients received primary and adjuvant radiotherapy, and elective neck dissection was performed more frequently. Age, sex, and adjuvant therapy did not affect survival, which remained stable compared to the earlier decade. However, lower stages of tongue cancer had a better prognosis when the tumour was located in the mobile tongue instead of the base of the tongue. In a further study from the same institution with 297 patients (1978-1987), Franceschi et al. demonstrated an improved overall 5-year survival rate of 65%, although the distribution of tumour stages was about the same compared to the preceding 10-year period [9]. Better survival was related to a more aggressive treatment of the neck even in early tumour stages and to adjuvant radiotherapy in advanced tumour stages. A considerable number of patients had to be upstaged after elective neck dissection due to occult lymph node metastases. The number of lymph node metastases turned out to be of prognostic value.

Since surgical treatment of tongue cancer strongly affects quality of life, many attempts have been made during the last decades towards organ preservation, leading to different treatment strategies with various combinations of surgery, radiation and chemotherapy [10]. Pernot et al. reviewed the medical records of 448 patients with tongue cancer who exclusively received radiation based therapy either as a combination of brachytherapy and external beam radiation or as a combination of brachytherapy and neck dissection [11]. The size of the lesion turned out to be the most important factor for prognosis with an overall 5-year survival rate of 44%.

The purpose of the present study was to give a precise description of our experience with surgical based therapy of tongue cancer during the last three decades. Furthermore, prognostic factors for survival were analyzed in order to obtain valid criteria for therapeutic decision-making in clinical routine.

## Patients and methods

Between January 1980 and December 2009, a total of 341 patients with squamous cell carcinoma of the tongue were treated at the Department of Oral and Maxillofacial Surgery, Hannover Medical School. Data concerning patient characteristics, clinical and pathologic tumour characteristics and treatment strategies and their results were obtained from a retrospective review of medical records. Informations regarding patient survival and local, regional and distant control were available for all patients. The average follow-up was 5.2 years. Statistical analysis for survival was calculated by the method of Kaplan and Meier. The relationship between the clinicopathologic variables and survival was assessed in univariate analysis using the log rank test. For multivariate analysis, the Cox proportional hazard model was used. A value of p ≤ 0.05 was considered to be statistically significant.

## Results

The average age at diagnosis was 58.8 years, ranging between 19.2 and 96.5 years. There were 226 men and 115 women (male/female ratio = 2/1). The primary site was the tip of the tongue in 8 cases (2.4%), dorsum of the tongue in 11 cases (3.2%), the base of the tongue in 91 cases (26.7%) and the lateral border of the mobile tongue in 231 cases (67.7%). There was a strong correlation between the primary site and the tumour size, with increasing tumour size towards the base of the tongue. Tumour extension across the midline was observed in 33 cases (9.7%). 14.5% of tumours were graded as well-differentiated, 69.6% as moderately-differentiated and 16.0% as poorly-differentiated. Anaplastic carcinomas were not observed. Nearly half of the patients suffered from T1-tumours (45.1%), followed by T2-tumours (32.7%) and T3- and T4-tumours (11.1% each). 309 patients received surgical treatment, whereas 32 patients were excluded from surgical treatment and received primary radio(chemo)therapy after biopsy. These patients refused surgery, were in inappropriate condition for general anaesthesia or suffered from inoperable tumour disease. As a consequence, the proportion of advanced tumour stages was higher in this group. Detailed information of histopathological and clinical staging results (pT-status/cT-status) of patients with and without surgical treatment are given in Table 1. Clinical staging results were based on recorded clinical examinations and - if present - evaluation sheets of ultrasound (US) and computed tomography (CT). Data from modern imaging techniques of initial clinical staging was almost complete for the second half of the investigation period. In patients with surgical therapy, the neck was staged pN0, pN1, pN2 and pN3 in 48.5%, 18.4%, 14.9% and 0.3% of cases. In 55 patients of this group (17.8%) the neck was staged pNx due to missing surgical therapy of the neck. At the time of diagnosis, lymphadenectomy was not considered necessary in these patients (cN0). In patients without surgical therapy, the presence of lymphatic spread was higher. In 10 patients of this group (31.3%) the neck was staged cNx due to missing clinical data. Detailed information of histopathological and clinical staging results (pN-status/cN-status) of patients with and without surgical treatment are given in Table 2. for both groups, there was a strong correlation between the tumour size at the primary site (T-status) and the presence of lymphatic spread (N-status). In the group of patients with surgical treatment, histologically assessed contralateral lymph node metastases were only observed in 9 patients (2.9%) of whom 5 patients had 1, 3 patients had 2 and 1 patient had 3 lymph node metastases on the contralateral side. Extracapsular spread was observed in 12.7% of patients with histologically assessed lymph node involvement, which strongly correlated with the degree of lymphatic spread (pN-status).

**Table 1:** Histopathological and clinical staging results (pT-/cT-status) of patients with surgical treatment and patients with radiotherapy

group	T-stage	n	%	valid%
	pT1	150	48,5	**49,0**
**surgical treatment**	pT2	108	35,0	**35,3**
	pT3	31	10,0	**10,1**
**(n = 309**)	pT4	17	5,5	**5,6**
	total	306	99,0	**100,0**
	missing	3	1,0	
	total overall	309	100,0	
	cT1	0	0,0	**0,0**
**non-surgical**	cT2	1	3,1	**3,7**
	cT3	6	18,8	**22,2**
**treatment (n = 32)**	cT4	20	62,5	**74,1**
	total	27	84,4	**100,0**
	missing	5	15,6	
	total overall	32	100,0	

**Table 2:** Histopathological and clinical staging results (pN-/cN-status) of patients with surgical treatment and patients with radiotherapy

group	N-stage	n	%
	pN0	150	48,5
	pN1	57	18,4
**surgical treatment**	pN2	46	14,9
**(n = 309)**	pN3	1	0,3
	pNx	55	17,8
	total	309	100,0
	cN0	0	0,0
**non-surgical treatment**	cN1	8	25,0
**(n = 32)**	cN2	13	40,6
	cN3	1	3,1
	cNx	10	31,3
	total	32	100,0

9.7% of the operated patients received neoadjuvant radiochemotherapy (30Gy/Cisplatin) prior to surgery, which was performed via a transoral (55.7%) or transmandibular approach (32.6%) or in pull-through technique (10.4%). 4 patients (1.3%) only received bilateral neck dissection, while the tumour at the primary site was radiated without surgery. Clear margins were achieved in 91.3% of the operated patients. On the ipsilateral neck, 44% of the operated patients received a comprehensive neck dissection, while 38.2% only received lymphadenectomy of level I-III and 17.8% no surgical therapy. On the contralateral neck, only 1.3% of these patients received a comprehensive neck dissection, while 36.6% still received lymphadenectomy of level I-III and the majority of 62.1% no surgical therapy. 19.5% of patients in the surgical group received postoperative radiation due to unclear margins, extensive tumour growth at the primary site, massive lymph node involvement or extracapsular spread, reflecting the scope of changing indications for radiotherapy during the past 30 years.

Local recurrence and regional recurrence were observed in 74 patients (23.9%) and 63 patients (20.4%) of the operated group, leading to total locoregional recurrence in 115 patients (37.2%) after surgical based therapy. In patients with regional recurrence, secondary lymph node metastases were located on the ipsilateral neck in 73.8%, on the contralateral neck in 18.0% and on both sides of the neck in 8.2%. Locoregional recurrence occurred after an average duration of 1.6 years after initial treatment. 10.9% of all patients (surgical plus non-surgical group) developed a second malignant disease during follow-up.

**Figure 1:**
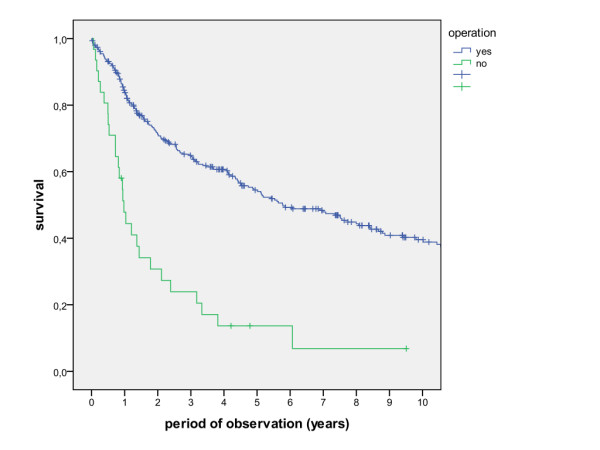
**Survival of patients with surgical treatment and patients with primary radio(chemo)therapy (log rank p < 0.001)**.

**Figure 2:**
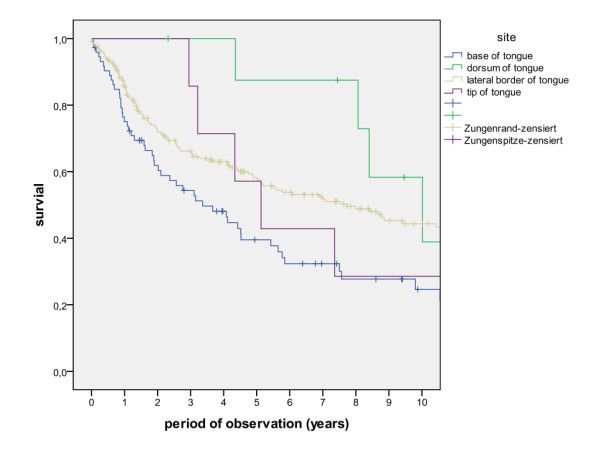
**Suvival of patients with different tumour sites (log rank p = 0.005)**.

**Figure 3:**
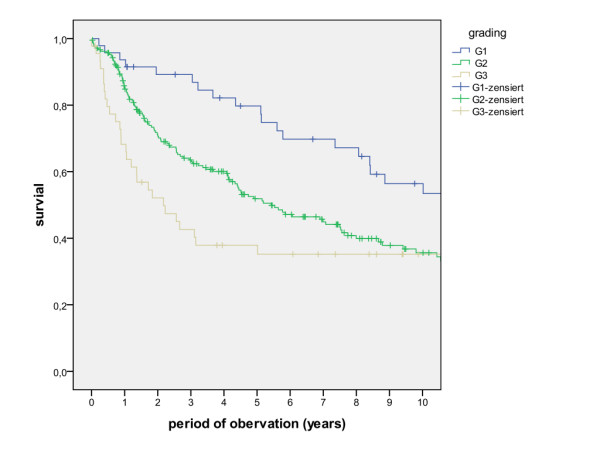
**Survival of patients with different tumour grading (log rank p = 0.004)**.

**Figure 4:**
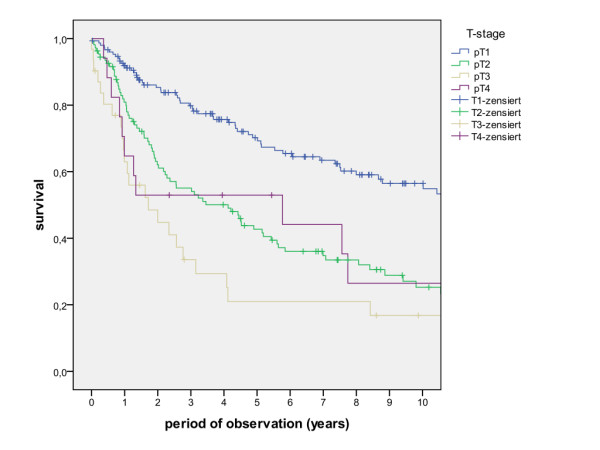
**Survival of patients with different pT-Stage (log rank p < 0.001)**.

**Figure 5:**
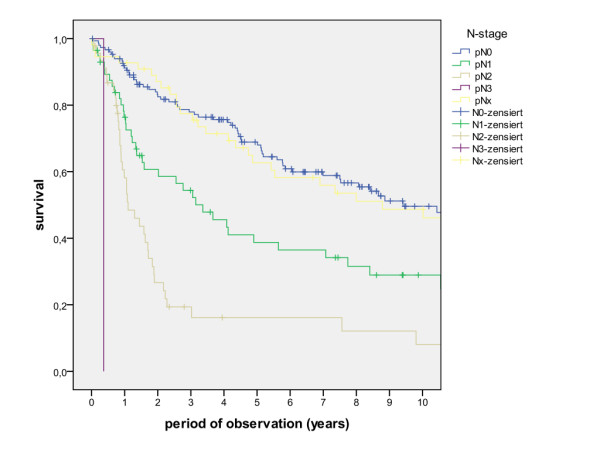
**Survival of patients with different pN-Stage (log rank p < 0.001)**.

**Figure 6:**
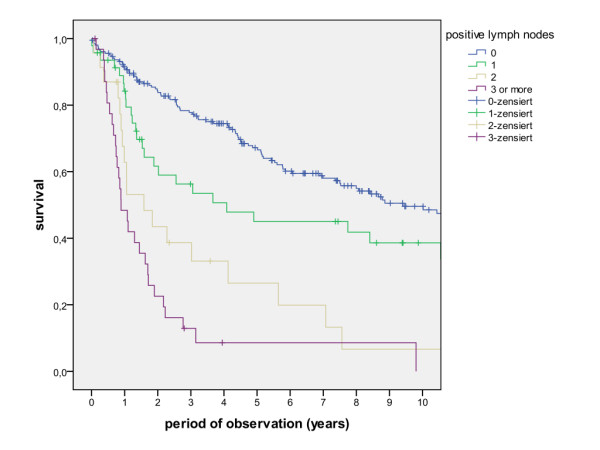
**Survival of patients with different numbers of positive lymph nodes (log rank p < 0.001)**.

**Figure 7:**
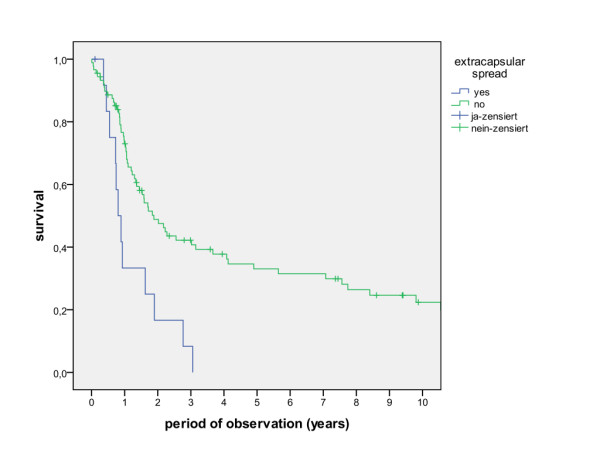
**Survival of patients with and without extracapsular spread of positive lymph nodes (log rank p < 0.001)**.

**Figure 8:**
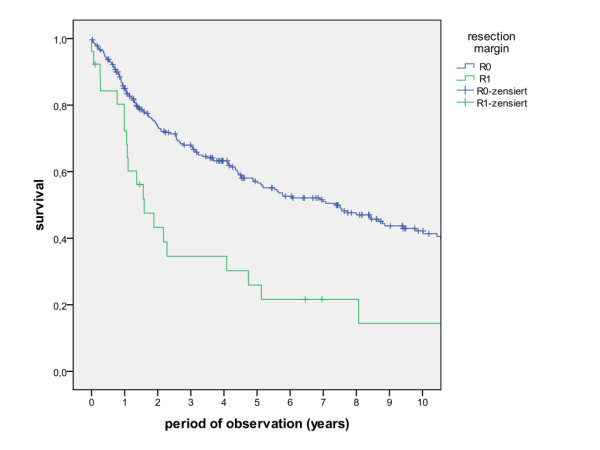
**Survival of patients with and without clear margins (log rank p < 0.001)**.

The overall survival rates after 1, 2, 5 and 10 years (including the surgical and non-surgical group) were calculated with 80.5%, 67.7%, 50.6% and 36.6%. The survival rates of the surgical group were calculated with 83.8%, 71.5%, 54.5% and 39.6%, whereas the survival rates of the non-surgical group were calculated with 47.8%, 30.7%, 13.7% and 6.8% (Figure 1, log rank p < 0.001). A detailed list of calculated survival rates for different T- and N-stages are given in Table 3 and Table 4. In univariate analysis (log rank), the following factors were identified as prognostic factors for survival after surgical based therapy: tumour site (Figure 2, p = 0.005), grading (Figure 3, p = 0.004), pT-status (Figure 4, p < 0.001), pN-status (Figure 5, p < 0.001), number of lymph node metastases (Figure 6, p < 0.001), extracapsular spread (Figure 7, p < 0.001) and clear margins (Figure 8, p < 0.001). Tumour extension across the midline (p = 0.356) and contralateral lymph node metastases (p = 0.922) did not show significant values. For N-status, extracapsular spread and clear margins, significant values were also confirmed in multivariate analysis (Cox proportional hazard model).

**Table 3:** Survival rates of patients with different pT-stages (surgical group)

pT-status	years	%
	1	91,9%
**pT1**	2	85,3%
	5	70,2%
	10	56,5%
	1	80,9%
**pT2**	2	62,1%
	5	42,7%
	10	25,2%
	1	63,0%
**pT3**	2	48,5%
	5	21,0%
	10	16,8%
	1	64,7%
**pT4**	2	52,9%
	5	44,1%
	10	26,5%

**Table 4:** Survival rates of patients with different pN-stages (surgical group)

pN-status	years	%
	1	91,2%
**pN0**	2	83,2%
	5	68,9%
	10	49,6%
	1	76,4%
**pN1**	2	60,7%
	5	38,7%
	10	24,8%
	1	58,1%
**pN2**	2	26,6%
	5	16,1%
	10	8,1%
**pN3**	1	0,0%
	2	0,0%
	5	0,0%
	10	0,0%
**pNx**	1	92,7%
	2	87,1%
	5	62,7%
	10	48,7%

**Figure 9:**
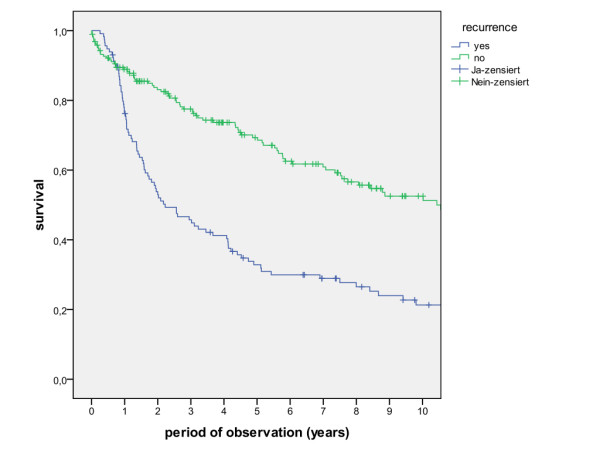
**Suvival of patients with and without locoregional tumour recurrence (log rank p < 0.001)**.

**Figure 10:**
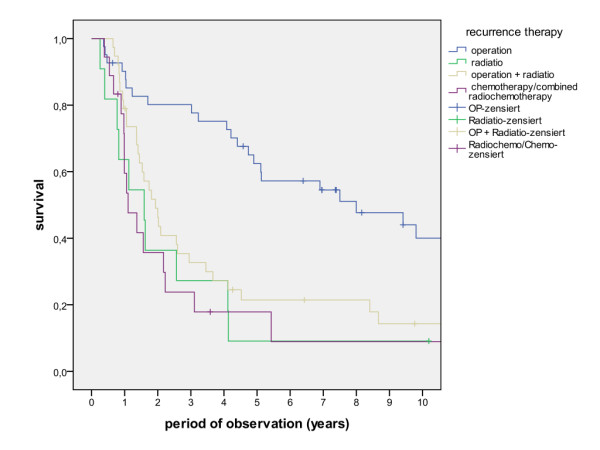
**Survival of patients with different treatment modalities for locoregional tumour recurrence (log rank p < 0.001)**.

Survival rates after 1, 2, 5 and 10 years for patients with and without local recurrence, regional recurrence and locoregional recurrence show significantly reduced survival for patients with tumour recurrence (Figure 9, log rank p < 0.001). Treatment modalities for tumour recurrence included singular surgery, surgery in combination with radiotherapy, singular radiation and combined radiotherapy, demonstrating a significant better prognosis when surgery was involved (Figure 10, log rank p < 0.001).

## Discussion

For many head and neck cancer patients, treatment consists of both chemotherapy and radiation therapy given simultaneously. This type of treatment is intensive and often results in serious and sometimes permanent damage to a patient's ability to swallow and hence, ability to eat a normal diet for the remainder of their lives. The locoregional recurrence rate of our patients lies within the range of locoregional recurrence rates decribed by other authors, which are quoted between 16 and 42% [12-17]. According to the literature, adjuvant treatment modalities seem to improve locoregional control. Patients undergoing a targeted chemora- diation protocol for head and neck cancer lost about 10% of their pretreatment weight and had a decline in eating ability [16,18]. In our study, almost 10% of the operated patients received neoadjuvant radiochemotherapy prior to surgery and almost 20% of patients in the surgical group received postoperative radiation due to unclear margins, extensive tumour growth at the primary site, massive lymph node involvement or extracapsular spread, reflecting the scope of changing indications for radiotherapy during a period of three decades. Since patient selection for (neo) adjuvant treatment was not randomized, the impact of radio(chemo)therapy could not be determined in our study.

Local failure at the primary site occurred in almost a quarter of our patients with surgical treatment, although clear margins were described for more than 90% of these patients. It is generally accepted that clear margins reduce local failure, although local control is not guaranteed. Byers et al. decribes local failure rates between 15 and 30% in patients with clear margins (> 5 mm distance to the tumour) and between 50 and 80% in patients with unclear margins [2]. In oncologic regard, it also remains unclear how much distance to the tumour should be maintained in tongue cancer.

The dimension of lymphatic involvement seems to reflect the degree of malignancy in tongue cancer [19-23]. In multivariate analysis, pN-status and extracapsular spread directly influenced survival besides clear margins. In univariate analysis, further factors showed prognostic value on the first sight. However, better survival of patients with tumours of the mobile tongue were attributed to the higher proportion of advanced tumour stages in patients with tumours of the base of the tongue, since these tumours were usually detected later [24,25]. The size of the tumour alone seemed not to be of relevance for prognosis as long clear margins were obtained. This also explains why tumour extension across the midline alone did not affect survival [26,27]. Furthermore, the statistical distribution of tumour grade and number of lymph nodes were also associated with prognostic factors which were later identified in multivariate analysis. Age and gender - as described by other authors - did not influence prognosis in our study [24].

In the literature, the prognostic value of tumour grade is controversially discussed. Whereas some authors consider tumour grade as a prognostic factor [24,26-29], other authors doubt the prognostic value of tumour grade [12,14,30-32]. It seems reasonable that there are further prognostic factors, which are still unknown and currently not detectable by modern imaging and histopathological techniques [33]. Therefore, a clear definition of high risk groups remains incomplete up to the present. In current literature, serum and saliva are considered very useful in the fields of genomics, proteomics, transcriptomics and metabolomics for generation of diagnostic and prognostic biomarker signatures [34-36]. However, first studies for oral cancer show that these techniques seem to have greater potential as a tumor diagnostic tool for follow-up than for prognostication [37]. Further validation by multi-institutional studies and randomized clinical trials are recommended before these techniques can be translated into clinical practice for oral cancer [38].

**Figure 11:**
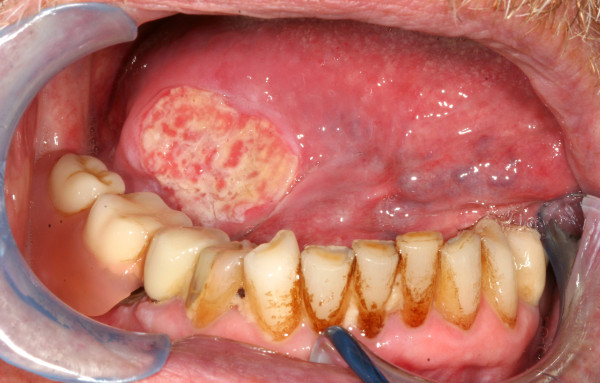
**Toung carcinoma defect of the right toung in a 64 year-old patient following resection of tumor. The reconstruction is planed with an anterolateral thigh flap (ALT-flap)**.

It is generally accepted that oral cancer and especially cancer of the tongue often shows lymph node involvement even in early stages [Figure 11]. The proportion of occult metastases is quoted between 24 and 42% [39-41]. The number of patients with initial lymph node involvement in our study was low compared to other studies [42]. Especially the number of patients with bilateral lymphatic spread was surprisingly rare, since bilateral lymph node metastases are observed more frequently by other authors [24,37,38]. This might be attributed to the reduced proportion of patients in our study with unilateral and especially bilateral neck dissection during initial treatment. However, a regional failure rate in every fifth patient is a clear indicator for too restrictive surgical management of the neck. The reduced survival rates of patients with regional failure shows that a "wait and see" policy on the neck is clearly not advisable. Many authors therefore recommend elective neck dissection even in early stages of tongue cancer when the neck is clinically staged N0 [17,39,40].

**Figure 12:**
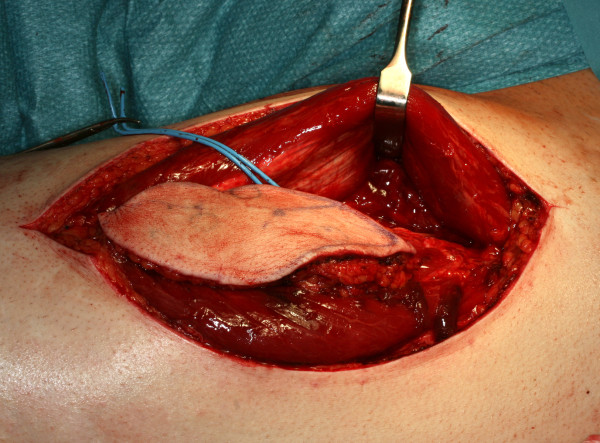
**Harvested anterolateral thigh flap (ALT-flap) based on the perforator vessels of the descending branch of the lateral circumflex femoral artery for reconstruction of the right toung**.

**Figure 13:**
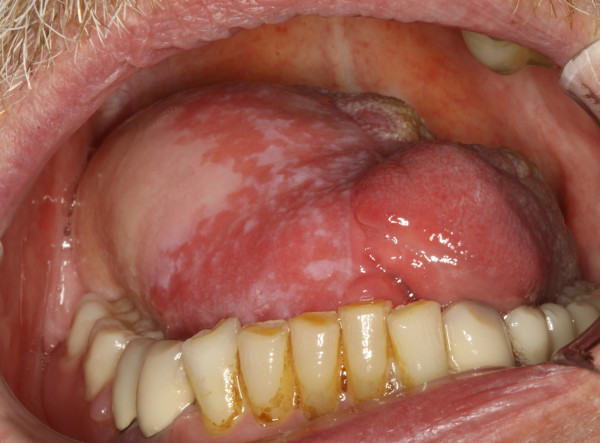
**Reconstructed defect of the toung. Final result of the anterolateral thigh flap (ALT flap) after 6 month**.

The overall survival rate of our patients with tongue cancer still lies within the range of survival rates decriped by other authors, which are quoted between 40 and 65% [7-9,11,24]. We have to keep in mind that our study reviews a period of three decades and that treatment strategies have changed during this period towards a more aggressive course [43,44]. On the base of our results with high locoregional recurrence rates even in early stages of tongue cancer, we generally recommend extended resections on the primary site and categorical bilateral lymphadenectomy of at least level I-III in order to reliably remove occult lymph node metastases which can not be detected even by modern imaging techniques. In case of an open staging procedure with histologically approved lymph node metastases during surgery, a comprehensive neck dissection should complete lymphadenectomy [45]. As described before, neck dissection procedures are only associated with a low morbidity [46]. Modern reconstructive techniques with microvascular tissue transfer [Figure 12] help to keep functional impairment after partial glossectectomy tolerable and at least allow to refill substancial loss of soft tissue after total glossectomy [47] [Figure 13]. According to our results, radical surgery also provides considerable survival rates for advanced stages of tongue cancer and should be recommended as treatment of first choice. Adjuvant treatment modalities should be applied more frequently in controlled clinical trials and should generally be implemented in cases with unclear margins and lymphatic spread.

In general, treatment strategies for tumour recurrence follow the same principles than for primary tumour disease. As already described by Eckardt et al., surgical intervention seems to be associated with better survival for patients with recurrent floor of mouth carcinoma, which was also confirmed by our study for patients with tongue cancer [48]. Therefore, curative total resection should be aimed if survival is clearly defined as highest preference. However, functional impairment with dramatic loss of life quality needs to be discussed individually with every patient. If recurrent tumour disease seems to be unresectable, subtotal tumour reduction can be discussed in order to improve the starting position for adjuvant treatment modalities in an interdisciplinary treatment concept. In general, however, mutilations caused by surgical interventions should be minimized in these cases in order to preserve the greatest amount of life quality as long as possible.

## Competing interests

The authors declare that they have no competing interests.

## Authors' contributions

HK and MR contributed equally to this work. JR, AE, FT, PS, DL, MRu and NCG conceived of the study and participated in its design and coordination. HK and MR drafted the manuscript. All authors read and approved the final manuscript.

## Acknowledgements

**Funding: **The article processing charges are funded by the Deutsche Forschungsgemeinschaft (DFG), "Open Acess Publizieren".
